# The immunological role of mesenchymal stromal cells in patients with myelodysplastic syndrome

**DOI:** 10.3389/fimmu.2022.1078421

**Published:** 2022-12-07

**Authors:** Likun Zheng, Lei Zhang, Yixuan Guo, Xintong Xu, Zhaoyun Liu, Zhenyu Yan, Rong Fu

**Affiliations:** ^1^ Department of Hematology, Tianjin Medical University General Hospital, Tianjin, China; ^2^ Department of Hematology, North China University of Science and Technology Affiliated Hospital, Tangshan, Hebei, China; ^3^ Department of Orthopedics, Kailuan General Hospital, Tangshan, Hebei, China

**Keywords:** myelodysplastic syndrome, mesenchymal stromal cells, immunomodulation, mesenchymal stromal cells-micro vesicles, immune dysfunction

## Abstract

Myelodysplastic syndrome (MDS) is a common hematological malignant disease, characterized by malignant hematopoietic stem cell proliferation in the bone marrow (BM); clinically, it mainly manifests clinically mainly by as pathological hematopoiesis, hemocytopenia, and high-risk transformation to acute leukemia. Several studies have shown that the BM microenvironment plays a critical role in the progression of MDS. In this study, we specifically evaluated mesenchymal stromal cells (MSCs) that exert immunomodulatory effects in the BM microenvironment. This immunomodulatory effect occurs through direct cell-cell contact and the secretion of soluble cytokines or micro vesicles. Several researchers have compared MSCs derived from healthy donors to low-risk MDS-associated bone mesenchymal stem cells (BM-MSCs) and have found no significant abnormalities in the MDS-MSC phenotype; however, these cells have been observed to exhibit altered function, including a decline in osteoblastic function. This altered function may promote MDS progression. In patients with MDS, especially high-risk patients, MSCs in the BM microenvironment regulate immune cell function, such as that of T cells, B cells, natural killer cells, dendritic cells, neutrophils, myeloid-derived suppressor cells (MDSCs), macrophages, and Treg cells, thereby enabling MDS-associated malignant cells to evade immune cell surveillance. Alterations in MDS-MSC function include genomic instability, microRNA production, histone modification, DNA methylation, and abnormal signal transduction and cytokine secretion.

## Introduction

Myelodysplastic syndrome (MDS) is associated with a poor clinical prognosis owing to its high risk of conversion to acute myeloid leukemia. It is, therefore, important that its pathophysiology be properly described for the development of effective treatment strategies. Mesenchymal stromal cells (MSCs) play an important role in the development and progression of the disease. This review describes the immune regulatory role played by these MSCs in patients with MDS, facilitating a wider and clearer view of the role played by these cells in the disease development and progression process, thereby identifying gaps in research on the subject and providing direction for future research. It also highlights the possibility of the use of MSC-based therapies for the treatment of MDS and other diseases.

## The myelodysplastic syndrome-related bone marrow microenvironment

Myelodysplastic syndrome (MDS) is a malignant hematopoietic stem cell (HSC) disease with a poor prognosis and a high risk of acute leukemia (1). Although demethylating agents and *BCL-2* inhibitors significantly improve remission rates in patients with this disease, most still relapse after 2–3 years, especially patients with high-risk MDS (HR-MDS); no clinically effective treatment is available for preventing disease progression to acute leukemia.

MDS prognostic score evolution and clinical typing have facilitated the identification of cytogenetic and epigenetic abnormalities, as well as primitive cells, associated with MDS development and prognosis. In addition, some researchers have found that *in vitro*, MDS-HSCs exhibit defective growth and differentiation, as well as poor transplantation capacity, in immunodeficient recipient mice (2). Cytogenetic and epigenetic abnormalities are commonly observed in several hematological disease states, and some researchers have even found that these epigenetic abnormalities can be easily detected in embryos (3–6). Thus, the mechanisms underlying MDS development and progression cannot be fully attributed to genetic and molecular changes alone. These findings suggest the existence of interactions between malignant cell clones and the bone marrow (BM) microenvironment, which includes immune cells and signaling pathways between cells, and this microenvironment may be one of the mechanisms of malignant cell cloning. Based on these findings, we can consider that the two main causative factors for MDS include stem cell abnormalities and microenvironmental disorders. MDS microenvironment abnormalities include abnormal morphology and functioning of MSCs, impaired differentiation of osteoblasts, increased number of vascular endothelial cells, abnormal number and functioning of immune cells, abnormal expression of cytokines, and abnormal activation of signaling pathways. Among these, MSCs and immune cells in the bone marrow microenvironment are the main factors in the pathogenesis of MDS.

**Figure 1:**
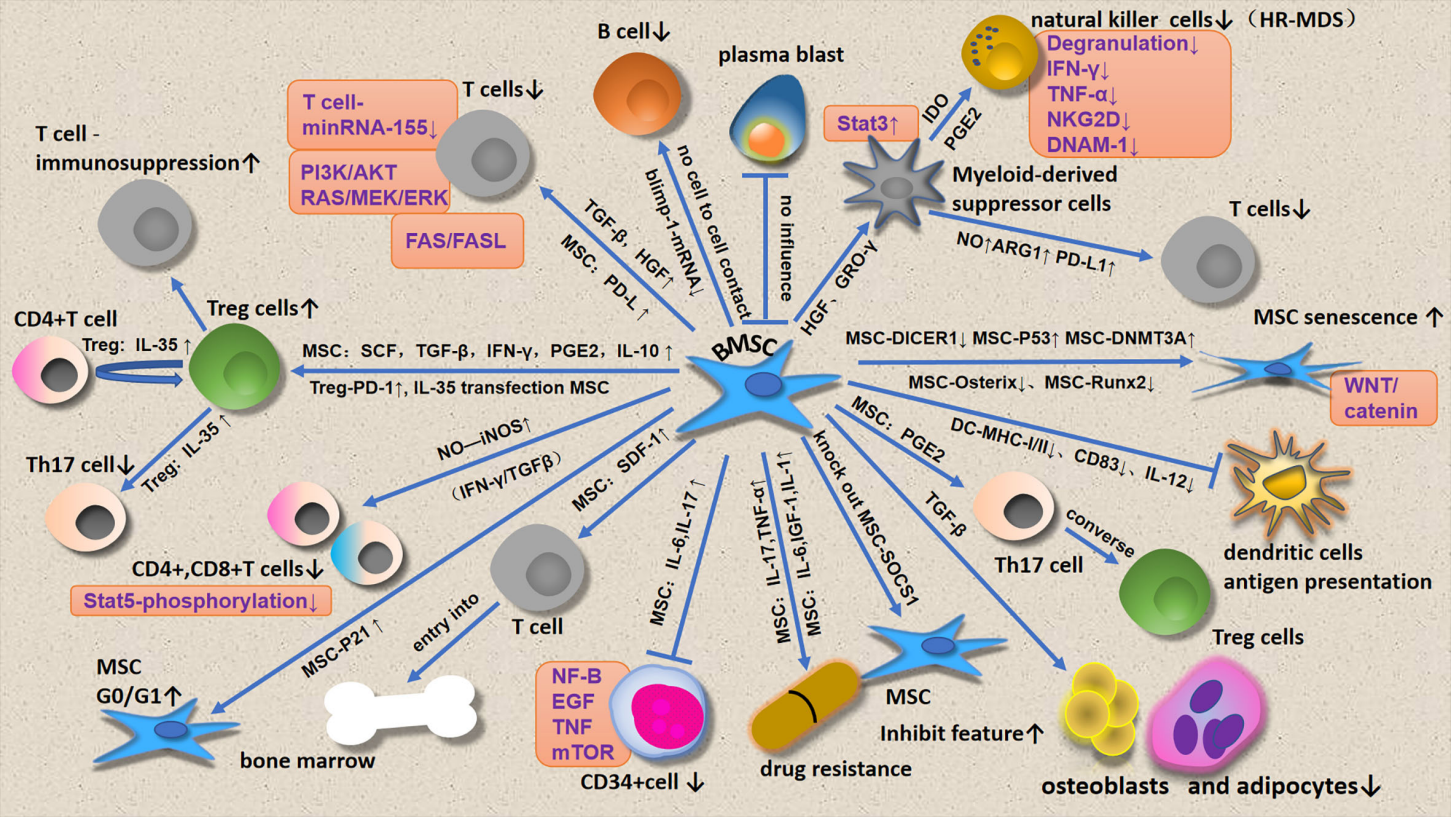
Effects of BMSCs on BM microenvironmental cells.

**Figure 2:**
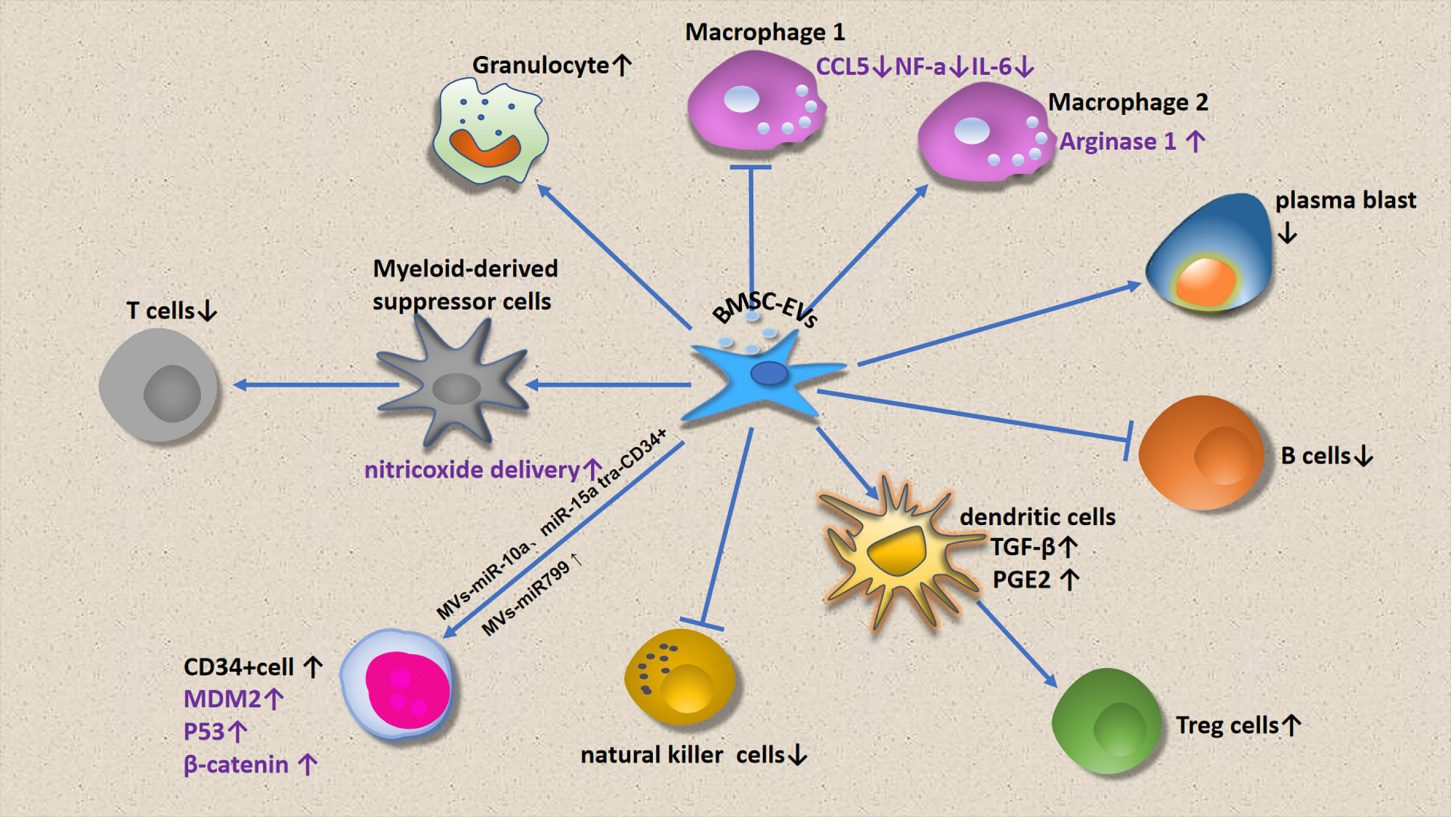
Effects of BMSC-EVS on BM microenviromental cells.

This review focuses on immune cell regulation by MSCs (**Figure 1**) and micro vesicles (**Figure 2**) secreted by MDS-associated bone mesenchymal stromal cells (MDS-MSCs), in patients with MDS. According to the findings of several studies, tumor growth, apoptosis evasion, drug resistance development, and tumor metastasis are inseparable from the tumor microenvironment (7, 8). Therefore, the abnormal BM microenvironment in MDS may serve as an umbrella to help tumor cells escape immune surveillance. The MDS BM microenvironment includes many cell types, such as MSCs, osteoblasts, fibroblasts, adipocytes, endothelial cells, immune cells, hematopoietic cells, extracellular matrix, cytokines, and MSC outer vesicles.

## Bone mesenchymal stromal cells in myelodysplastic syndrome patients

### Mesenchymal stromal cell properties

Friedenstein et al. were the first to discover and describe MSCs (9). The sources of MSCs are rich (10), including BM, fat, synovial membrane, bone, muscle, lung, liver, pancreas, and cord blood, among which BM-derived MSCs mainly originate from endothelial cells (11), but their number is very low (0.001–0.01%) (12).

MSC separation and expansion are easy to perform; besides, their cell properties are stable after expansion, and MSCs have homing to inflammatory sites, multidirectional differentiation ability, and immunoregulatory properties; they can also regulate the proliferation and differentiation of HSC by secreting E-selectin (13), cytochemokines, and crosstalk molecules, such as Jagged1 and CXCL12 (14–16), and this regulation also depends on the tight spatial localization of MSC and vascular endothelial cells (17).

After culture, MSCs are typically observed as a mural spindle; 95% of the cells express CD73, CD105, and CD90, and less than 2% of the cells express CD14, CD34, CD45, CD79a, and HLA-DR. These cells can differentiate into osteocytes, chondrocytes, adipocytes, tenocytes, and myocytes (9). Thus, MSCs have been clinically applied in regenerative medicine and immune disease treatment (18).

### MSCs in myelodysplastic syndrome patients

Reported *in vitro* data have indicated the lack of difference in phenotype and growth characteristics between MSCs in all MDS subtypes (19). A few studies have shown that mesenchymal cell counts are not reduced in patients with MDS (20). However, it has been confirmed in several reports that the phenotype and function of MSCs are significantly altered in acute myelogenous leukemia (AML) and late-stage (high-risk) MDS, but not in early-stage (low-risk) MDS (21, 22), indicating that MSC damage and disease severity are correlated in MDS.

MDS-associated MSCs exhibit increased senescence rates, manifested by decreased proliferative capacity and increased expression of P53 and P21, which regulate cellular senescence; P21 was found to arrest cells in the G0/G1 phase (23). This decrease in MSC proliferative capacity may be related to chromosome methylation status, an abnormal proliferation regulation signaling pathway, and cell cycle arrest, among other factors (24, 25). As expected, a number of studies have reported increased levels of MDS-associated MSCs senescence markers, including reduced telomere lengths and increased β-galactosidase levels (26), compared with those in healthy MSCs. However, the *Osterix* and *Runx2* genes were found to be downregulated in MDS-MSCs (27). The downregulation of DICER1, an RNase III enzyme involved in microRNA biogenesis, was found to be implicated in increased MDS mesenchymal progenitor cell senescence (28). Increased *DNMT3A* expression levels in MDS-derived MSCs, which leads to the hypermethylation of specific genes, is significantly associated with MSC aging (29). MDS-MSC aging results from both classical Wnt/catenin signaling and non-classical signaling. Decreased Wnt/catenin classical signaling pathway activity is significantly correlated with decreased MDS-MSC proliferation and differentiation potential (25). The activation of NF-κB mediated cellular stress and MSC proliferative damage (30).

TGF-βmiRNA is overexpressed in CD34+ cells in patients with MDS; TGF-β can inhibit MDS-MSC function (26). In addition, it can significantly reduce the MSC differentiation potential in relation to osteoblasts and adipocytes (31, 32). An increase in endogenous erythropoietin level is often observed in patients with MDS, which may downregulate the Wnt pathway and further impair MDS-MSC osteogenic differentiation (33). Some scholars have shown that the immunoregulatory function of MSCs in patients with MDS is damaged (34, 35), while other scholars have demonstrated that there is no significant difference in the immunosuppressive function between MSCs of healthy individuals and patients with MDS (36). In addition, MDS-associated MSCs exhibit their immunosuppressive effects by increasing prostaglandin production, which may reduce the destruction of leukemic cells by T cells (37). Furthermore, Zhang et al. demonstrated that treating MSCs with inflammatory cytokines could induce the production of cytokine signaling inhibitory molecule 1 (SOCS1) by MSCs, and SOCS1 knockdown was found to enhance the immunosuppressive capacity of MSCs (38).

In patients with MDS, MSCs show different genetic abnormalities compared with HSCs (39). As compared to MSCs derived from healthy individuals, MDS-associated MSCs appear to be more susceptible to acquiring mutations during culture. Some researchers have found that MDS-associated MSCs had higher levels of genotoxic stress markers, for example, the frequency of γH2AX foci phosphorylation or replication protein A (RPA) phosphorylation, than those of healthy MSCs (40). In MDS, γH2AX staining findings were found to correlate with mutation frequency. The occurrence of myelodysplastic changes and common genetic changes in AML are associated with A β-catenin-activated MSC mutations (41). The activation of β-catenin induces MSCs to express Jagged1, triggering the activation of notch signaling in hematopoietic stem progenitor cells (HSPCs) and subsequently promoting the progression of leukemia. Highly purified CD271+ MSCs isolated from low-risk MDS (LR-MDS) patients have the ability to fully activate the inflammatory response, which involves NF-κB, EGF, TGF-β, and TNF signaling (42).

### The role of MSCs in the onset of MDS

**Figure 3:**
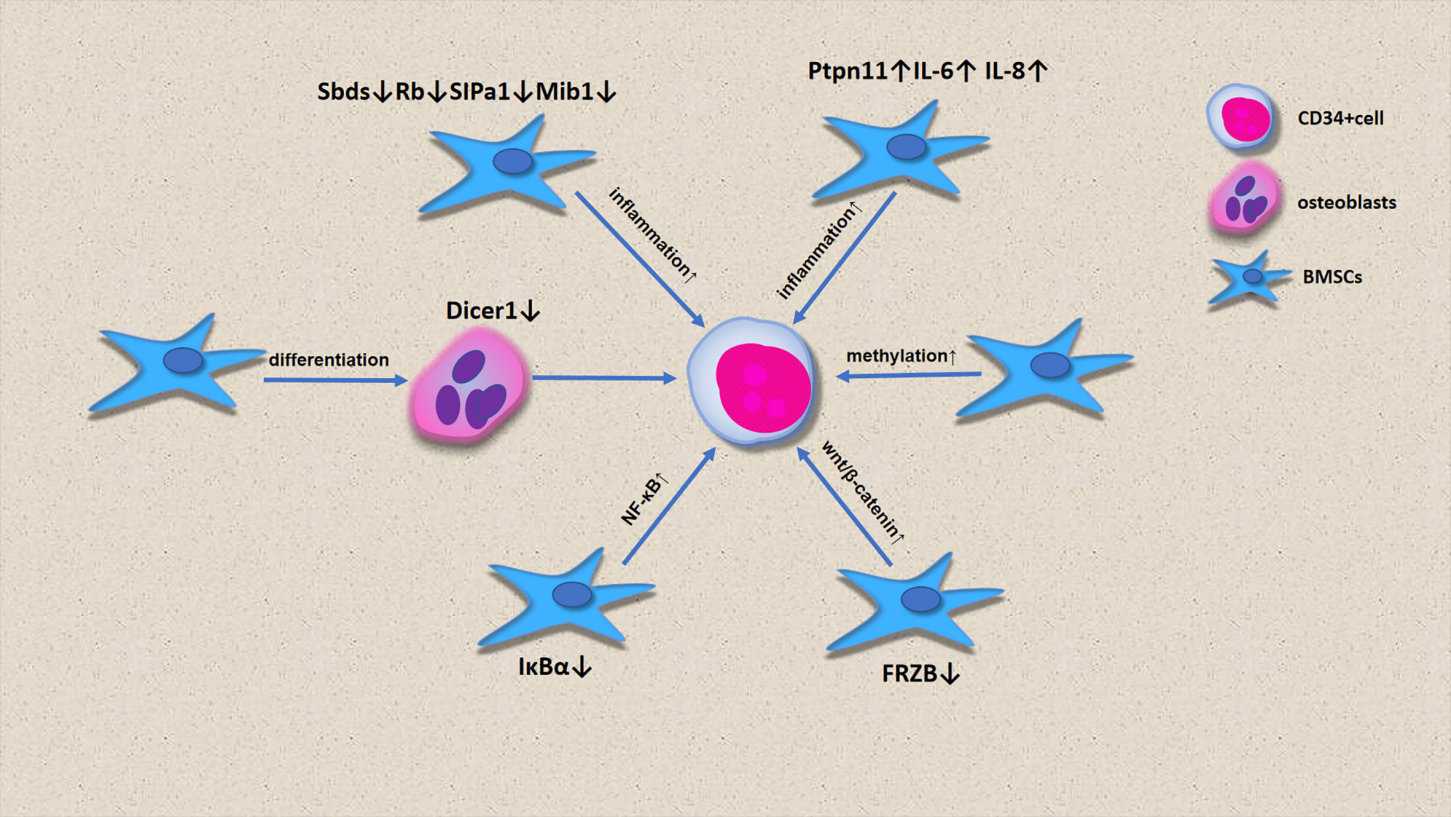
The role of MSC in the onset of MDS.

In the past, most scholars believed that MDS was caused by HSC lesions. However, increasing evidence has shown that microenvironment defects can also lead to ineffective hematopoiesis, which leads to the onset of MDS and even progression to AML (**Figure 3**). MSCs in microenvironments are considered to be substrates with no clear function. Bone cells differentiated by MSCs participate in the hematopoietic stem cell niche, and selective deletion of DICER1 in bone progenitor cells can induce hematopoietic disorders, which is one of the pathogenetic characteristics of MDS (43). Downregulation of DICER1 expression in MSCs was also observed in patients with MDS (28). However, the downregulation of SBDS, Rb, SIPA1 and MIB1 expression and the upregulation of PTPN11, IL-6 and IL-8 expression by BM-MSCs promotes an inflammatory environment in the bone marrow, enabling the onset of MDS (42, 44). CD34+ cells from healthy people had poor colony formation after co-culture with BM-MSCs. Contrastingly, CD34+ co-culture with BM-MSCs with 5-Aza led to an increase in the number of hematopoietic colonies, indicating that MSCs from MDS show high methylation, which is involved in the pathogenesis of MDS. FRZB is an antagonist of the Wnt pathway, and the downregulation of FRZB in MSCs leads to β-catenin activation, which promotes the onset of MDS, and this is more obvious in HR-MDS (45). lκBα is an inhibitor of NF-κB. The lack of IκBα or activation of NF-κB in hematopoietic cells is not sufficient to induce abnormal cell development, but the imbalance of NK-κB in non-hematopoietic cells leads to the onset of MDS (30, 46).

## Effects of MDS-MSCs on HSC growth and survival

A study found that when healthy hematopoietic cells are exposed to MDS-associated MSCs, they will have a toxic effect on CD34+ HSPCs, and this effect is persistent, especially in high-risk patients, making this phenomenon is more evident (47). Such abnormal MDS-MSCs may not be able to support normal hematopoiesis, and may also affect the clonal proliferation of HSCs (31, 48). Other studies have found that MSC-micro vesicles (MSC-MVs) exert hematopoiesis-regulating effects. MDS-MVs are generated by a direct blossoming from the plasma membrane.

The number of pluripotent HSCs is generally low. These HSCs are at the top of hematopoietic cell development and have the ability to replicate and renew themselves to differentiate and replenish blood cells of various lineages (49). During differentiation, HSC progenitors undergo different developmental stages, including multi-potential progenitor cells and progenitor cells of various lineages (50). The BM contains niches that protect stem cells. Under normal circumstances, HSPCs are located in these niches and rarely appear in the peripheral blood (PB). MSCs derived from both early-stage (51) and late-stage patients with MDS (52) were found to exhibit decreased expression of ANGPT, which functions to maintain HSPC quiescence (48). Furthermore, we found changes in the expression levels of key molecules involved in MSC interaction with HSPCs in patients with MDS, particularly, osteopontin, Kit-ligand, Jagged1, and angiopoietin, as well as several chemokines. The change of MDS-MSCs significantly reduced the hematopoietic support ability of CD34+ HSPCs, which was related to the reduction of cell cycle activity. MSCs promote MDS development by creating an inflammatory environment. The findings of some studies have shown that IL-6, IL-17A, IFN-γ, and TNF-α produced by MSCs may contribute to HSPC dysregulation, leading to ineffective hematopoiesis (53). In patients with MDS, the inflammatory pathways of MSCs are overactivated, and these pathways include the NF-B, EGF, TGF-β, and TNF-α, which hinder hematopoiesis in LR-MDS; this is considered to be responsible for the increased apoptosis rates observed at this stage of the disease (30, 42). MDS-MSCs exhibit hypermethylation, and treatment of MDS-MSCs with demethylated drugs can improve the formation of HSC colonies (54).

However, miR-7977 upregulation in micro vesicles secreted by MSCs promote CD34+ cell proliferation. Increased secretion of miR10a- and miR15a-containing micro vesicles by MDS-MSCs increases the viability and clonogenicity of HSPCs in patients with MDS (55). Transfection of miR-10a- and miR-15a-containing micro vesicles secreted by MSCs into CD34+ cells induced an increase in their activity; in addition, these cells exhibited increased *TP53* and *MDM2* expression (55). Another study showed that MSC-MVs increase β-catenin expression in expanded CD34+ cells (56); furthermore, the loss of β-catenin has been shown to impair the self-renewal ability of HSCs (57). MSC-MVs possibly exert their hematopoiesis-supporting effects through the Wnt/β-catenin pathway.

HSC mainly exists in the perivascular niche. Endothelial cells (ECs) are an important component of the BM microenvironment and participate in the proliferation of HSCs. ECs in patients with MDS can be mobilized from BM to become circulating progenitors of ECs (cPECs). Patients with MDS who have increasing numbers of cPECs have increased methylation levels, which affects HSC function (58). Adipocytes, which are also components of the BM microenvironment, are negative regulators of HSC, and the number of adipocytes in BM is negatively correlated with the number of HSCs (59). Another major component of the BM microenvironment is osteoblasts, but current studies have shown no significant association between osteoblasts and HSCs. Therefore, it is not clear whether osteoblasts have a regulatory effect on HSCs (60).

## MDS and MDS-MSC drug resistance

The treatment of MDS mainly includes blood transfusion support, iron removal therapy, demethylation therapy, targeted therapy for mutated genes, and BM transplantation. However, the overall therapeutic effect is not ideal, and the long-term survival rate is still less than 30%; thus, targeted BM matrix therapy is attracting researchers’ attention. New areas of research also include gene -editing MSCs (61) and nanoparticle-coated small interfering RNA (siRNA) (62).

In patients with MDS, MSCs may improve the drug resistance of CD34+ cells through two factors: endogenous factor and exogenous factor. Endogenous factor-induced drug resistance is thought to be triggered by preexisting intrinsic random gene mutations in CD34+ cells (63–65). As opposed to extrinsic factors, such as environment-mediated drug resistance (EM-DR), extrinsic factors may not only protect tumor cells with genetic mutations, but also trigger the development of other mutations. EM-DR is induced by signaling events triggered by certain factors present in the tumor microenvironment. EM-DR is divided into two categories: soluble factor-mediated drug resistance (SFM-DR) and cell adhesion-mediated drug resistance (CAM-DR) (66–68). The mechanisms of SFM-DR and CAM-DR are different. The former is mainly mediated by inducible gene transcription, while the latter is mainly mediated by non-transcriptional mechanism (68), including degradation of apoptotic activators and increased stability of apoptotic inhibitors and cell cycle regulators (69).

BM-MSCs are highly expressed in cytidine-deaminase (CDA), which can metabolize two essential drugs for MDS treatment: azacitidine and decitabine (70). BM-MSCs can also increase drug resistance by silencing CD34+ cells, thereby protecting CD34+ cells from apoptosis. In addition, MSCs can inhibit the apoptosis of CD34+ cells and promote their proliferation by activating the mTOR signaling pathway (71). *In vivo*, MDS-MSCs may transfer functional mitochondria to CD34+ cells, thereby increasing the survival of CD34+ cells in the presence of chemotherapeutic conditions (72, 73).

Drug-resistant malignant tumor cells highly express very late antigen4 (VLA4), which enables malignant tumor cells to attach to MSCs *via* VCAM1, thereby activating the NK-κB signaling pathway (74). Tumor cells with negative expression of VLA4 were more responsive to treatment, suggesting an association between the development of drug resistance and MSCs. MSCs express CXCL12. Thus, connecting CXCR4 with HSCs and blocking the CXCL12-CXCR4 axis can make tumor cells sensitive to chemotherapy drugs (75, 76).

## Immunomodulation of the bone marrow microenvironment by MDS-MSCs

In patients with MDS, MSCs also exhibit immunosuppressive function; MDS-MSCs secrete high levels of TGF-β1 (77), a cytokine that exerts significant immunosuppressive effects on B, T, and natural killer (NK) cells, as well as immunostimulatory effects on regulatory T cells (Treg cells). Thus, the high TGF-β1 expression levels observed in patients with HR-MDS (77) promote the development of an immunosuppressive microenvironment, characterized by a decrease in the CD4+ T-cell population, CD8+ T-cell exhaustion, decrease in NK-activating receptor expression, and increase of non-cytotoxic NK-cell counts (CD56bright) (78). MSCs exert significant immunosuppressive and anti-inflammatory effects through several contact-dependent and contact-independent mechanisms (79).

### Effects of MDS-MSCs on T lymphocytes

MSCs affect T cells in several ways, including inhibiting T-cell proliferation and cytokine secretion and cytotoxicity, as well as regulating T helper (Th)1/Th2 effector cell balance (80).

Increased SDF-1 expression in MDS-MSCs may induce circulating T-cell entry into the BM by chemotaxis (31). Zhao et al. found that the immunosuppressive effects of high-risk MDS-MSCs on T-cell proliferation were significantly higher than those of low-risk MDS-MSCs (77). The findings of *in vitro* experiments have confirmed that the effects of high-risk MDS-MSCs in inhibiting effector T-cell proliferation, which are mediated by the promotion of TGF-β production and secretion, are significantly higher than those of low-risk MDS-MSCs (77). Most scholars believe that MSCs exert their immunosuppressive effect only under the mediation of certain factors, which are mediated by the upregulation of T-cell suppressive pathways, until they are activated by proinflammatory cytokines, especially IFN-γ (81). Notably, in some studies, MSCs have been found to play no significant immunomodulatory role in the absence of IFN-γ or other inflammatory signaling molecules (82). MSCs induce activated T-cell anergy through the production of IL-10, which enhances regulatory T-cell development, thereby inhibiting conventional T-cell proliferation and the activities of other effector cells, MSC-based indoleamine 2,3-dioxygenase (IDO)-dependent immune modulation mechanisms (83), as well as the downregulation of costimulatory molecules, CD40, CD80, and CD86 (84), which are dominant in humans. MSCs can promote nitric oxide synthase synthesis (NOS) and inhibit STAT5 phosphorylation in T cells, thereby inhibiting their function (85). A net loss in activated T cells through an increase in cell death can lead to immunosuppression over time (86). In addition, the effect of MSCs on activated cytotoxic T cells (CTLs) is mainly manifested in the inhibition of their lytic function (87).

Besides, MSCs were also found to alter Th1, Th2, Th17, and Treg cell secretory function through the secretion of soluble cytokines, exosomes, and micro vesicles.

MSC-induced T-cell immunosuppression has been extensively studied; this MSC-induced T-cell immunosuppression, which can alter the Th1/Th2 cell ratio by secreting soluble cytokines, TGF-β, stem cell growth factor (SCF), prostaglandin E2 (PGE2), and INF-γ, induces an increase in Treg cell counts.

Aside from the soluble factors mentioned above, MSCs can also downregulate the activation and responses of T cells by interacting with T cells; this occurs by inhibiting the interaction of the receptor programmed cell death-1 (PD-1) protein with its cognate ligands PD-L1 and PD-L2 to induce apoptosis of T cells (88). miR-155 downregulation in T cells was found to inhibit T-cell proliferation. According to scholars, the reason for the immunosuppression of T cells by MSCs is not to induce T-cell apoptosis, but to arrest T cells at the G0/G1 phase of the cell cycle (89). The effects of MSCs on T cells involve changes in several signaling pathways, such as the PI3K-AKT, RAS-MEK-ERK, STAT3 phosphorylation, FAS/FASL, and WNT/-catenin pathways, which are all involved in this process. MSC-MVs were found to inhibit T lymphocyte proliferation, and these effects were more significant in the MSC-MV group than in the MSC group (56).

### Effects of MDS-MSCs on B lymphocytes

The inhibition of B cells by MSCs does not require cell-cell contact, but is mediated by humoral factors secreted by MSCs; in addition, it is associated with decreased Blimp-1 mRNA expression. Unlike T cells, IL-10, TGF-β, and IDO are not involved in the inhibition of B cells by MSCs. MSCs increase the expression of IgG3 in B cells. Further, MSCs could inhibit the proliferation of B cells induced by lipopolysaccharide (LPS), but had no effect on plasma cell apoptosis. Plasmablast generation was found to be inhibited in the presence of MSC-derived extracellular vehicles (EVs) (90). *In vitro*, MSC-EVs preferentially select B lymphocytes cells when binding to immune cells (90).

### Effects of MDS-MSCs on NK cells

NK cells exert their intrinsic antitumor activity by lysis of defective cells. However, MDS-MSCs desensitize this NK cell activity.

In HR-MDS, the number of NK cells decrease, allowing the proliferation of malignant clones. However, NK cells appear to be cytotoxic toward malignant clones, thereby slowing disease progression (91–94).

When cultured *in vitro*, MSCs from patients with MDS and healthy donors have similar phenotypes, and co-culture with NK cells did not affect NK cell function. However, when MDS-MSCs and HD-MSCs were co-cultured with monocytes, only MDS-MSCs could induce monocytes to possess the phenotypic and metabolic characteristics of myeloid-derived suppressor cells (MDSCs), thereby inhibiting NK cell function. In addition to inhibiting NK cell degranulation and proliferation, monocytes under the MDS-MSC condition also inhibited the production of IFN-γ and TNF-α of NK cells, which was similar to the situation of NK cells co-cultured with MDSCs *in vitro* (95). Carlsten et al. reported that MDS-MSCs played a role in suppressing the innate immune system by inducing monocytes, while HD-MSCs did not. The key receptors controlling human NK cell self-recognition are HLA class I-binding receptors, which include the killer immunoglobulin-like receptor (KIR) family, natural killer group 2A (NKG2A) receptors, and leukocyte immunoglobulin-like receptor subfamily B member 1 (LILRB1, also known as LIR-1) (96). Several studies have confirmed that NK cells decrease NKG2D and DNAM-1 expression, especially in patients with HR-MDS (97). MSCs impair resting NK cell cytolytic activity through the production of IDO and PGE2 (98). Carlsten et al. further reported that *in vitro*, NK cells lose their potent anti-tumor properties, as indicated by impaired cytotoxicity to CD34+ MDS blast cells, resulting in enhanced tumor evasion. MSC-MVs were found to inhibit NK cell proliferation, and these inhibitory effects were more significant in the MV group than in the MSC group (56).

### Treg cell and MDSC regulation by MDS-MSCs

Treg cells play an immunomodulatory role by suppressing abnormal or excessive immune responses, either to self- or non-self-antigens, thereby maintaining immune homeostasis. Treg cells inhibit the antitumor immune response and participate in the occurrence and progression of tumors. They also play separate and unique roles in LR-MDS and HR-MDS. In LR-MDS, T-cell activation and apoptosis were not excessively inhibited due to the small number of Treg cells. However, the number of Treg cells is increased in HR-MDS, which inhibits the endogenous immune system ability to clear malignant clonal cells, and thus, malignant clonal cells proliferate (91). Zhao et al. reported that HR-MDS-MSCs could induce CD4+CD25 T cells to transform into CD4+CD25+Foxp3+Treg cells, and the induction rate was significantly higher than that of LR-MDS-MSCs, and the induced CD4+CD25+Foxp3+Treg cells caused T-cell inhibition (77). In late-stage MDS, anti-leukemic immunity is decreased, which may be related to Treg cell expansion at this stage (99), which is also associated with higher BM blast infiltration, higher IPSS scores, and disease progression (100). MSCs can secrete PGE2, which regulates the conversion of Th17 to Treg cells (101). MSCs can induce Treg cell expansion *via* IL-10 secretion. IL-35 is secreted mainly by Treg cells, and is an effector molecule through which these cells exert negative immunoregulatory effects; IL-35 can induce CD4+ T-cell differentiation into Treg cells with immunosuppressive functions. In addition, IL-35 inhibits Th17 proliferation and differentiation. Furthermore, IL-35-transfected MSCs were found to induce an increase in Treg cell counts (102). MSC-exposed Treg cells exhibit increased immunosuppressive activity; this effect may be related to the activation of PD-1 receptor on Treg cell membranes and the production of IL-10 in the MSC/Treg co-culture system (103). Moreover, MSC-mediated Treg cell function is related to PGE2 and MSC TGF-β, as well as contact-dependent mechanisms (104).

MDSCs are derived from immature myeloid cells and have the ability to inhibit immune cell response. The number of such cells is remarkably increased in patients with tumors; this accelerates tumor progression (105–107). This immunosuppression in patients with cancer is associated with a variety of factors, such as concentrations of soluble factors with immunosuppressive activity, loss of effective antigen presentation, abnormal effector cell function, and the recruitment of immunosuppressive cell populations, such as MDSCs (108, 109). The number of MDSC cells in the PB and BM of patients with HR-MDS were significantly higher than those of patients with LR-MDS (110, 111). In tumors, MDSC contributes to the formation of immune tolerance microenvironment, in which MSC promotes the expansion of MDSC (80). Moreover, the expression levels of signal transducer and activator of transcription 3 (STAT3) and C-C chemokine receptor type (CCR) 2 on MDSCs were increased in HR-MDS. Targeted inhibition of the STAT3 pathway reduced ARG1 expression in MDSCs and partially reverses the decrease in effector molecules in CD8+ T lymphocytes (112). It has been confirmed that MSCs in the BM microenvironment promote the expansion and activation of MDSC by secreting hepatocyte growth factor (HGF), which also involves the activation of the STAT3 pathway (113). Moreover, MDSCs not only inhibited T-cell activity, but also induced CD4+CD25highCD127low Treg cells, thus further improving immunosuppression (113). The growth-regulated oncogene (GRO) chemokine GRO-γ, secreted by MSCs, induced the expansion of MDSC. The transformation of monocyte-derived dendritic cells into MDSC was induced when GRO-γ was added to MSC medium (106). BM-MDSCs inhibit T-cell function without close contact between MDSCs and T cells; this involves increased expression levels of nitric oxide, ARG1, and immunosuppressive cytokines (114–116). MDSCs secrete immunosuppressive cytokines, thereby reducing effector T-cell proliferation. In addition, there is usually an increase in MDSC counts in the BM in MDS, and the magnitude of the abundance of MDSCs is a poor prognostic indicator in such patients. PD-L1 expressed on MDSC membranes binds to T-cell-expressed PD-1, thereby inhibiting T-cell function (117, 118). BM-MSC-derived exosomes were found to stimulate MDSCs *in vivo*, thereby increasing their nitric oxide delivery capacity, in which nitric oxide participates in T-cell blockage (106).

### Effects of MDS-MSCs on macrophages

Macrophages interact with and regulate HSCs (119). They also provide critical support for erythropoiesis in the BM erythroblastic islands (120). Macrophages produce vascular endothelial growth factor (VEGF), which stimulates angiogenesis. Studies have shown increased blood vessel density in the BM in patients with MDS, and macrophages may play an indirect role in disease development (121). Some studies have found that the action of pro-apoptotic cytokines causes an increase in the number of macrophages in the BM in MDS (122). Other scholars found that macrophages were inhibited in MDS, and this was more evident in HR-MDS (123). Notably, hematopoietic precursor cell apoptosis is a hematopoietic feature of LR-MDS (124). As macrophages are responsible for engulfing apoptotic cells in the BM (125, 126), they may play a particularly important role in LR-MDS. However, the alteration of macrophage phagocytic capacity MDS has not been determined. In a mouse stem cell transplantation model, macrophages were important for successful HSC implantation and growth, suggesting their role in maintaining BM homeostasis and normal hematopoiesis (91). Further, a study showed that EVs can affect macrophage maturation, inducing lower TNF-α and higher IL-10 expression levels in these cells (127). Another study showed that MSC-EVs decrease the expression levels of several proinflammatory signaling molecules secreted by M1 macrophages, such as CCL5, TNF-α, and IL-6, while promoting the expression of the M2 macrophage-derived immunomodulatory factor, arginase 1 (128).

### Effects of MDS-MSCs on dendritic cells

Dendritic cells are specialized in terms of processing and extracting various antigenic substances. They are antigen-presenting cells and are crucial for T-cell activation. MDS-associated DCs become dysfunctional, impairing immune responses (129). In addition, decreased DC counts have been observed in MDS states, with this decrease being more significant in HR-MDS than in LR-MDS (130). MSCs can impair DC antigen presentation by inhibiting the expression of MHC I and II or costimulatory proteins (such as CD83), or inhibiting the production of IL-12 (131, 132). Studies have shown that MDS-associated DCs can activate endogenous T cells; furthermore, it has been reported that the number of DCs are reduced in MDS, and DCs show defects in the activation effect of Treg cells (133). This finding is supported by data from recent *in vitro* studies. Analysis of DCs isolated from MDS (MDS-refractory anemia and MDS-refractory anemia with ringed sideroblasts) showed a significant reduction in the number of mature and immature DCs and an inhibition of the ability of DCs to present antigen to Treg cells (134), highlighting a significant opportunity for therapeutic intervention (130). During DC and naïve T-cell co-culture, MSC-EV-stimulated immature DCs were found to produce immunomodulatory factors, such as TGF-β and PGE2, thereby inducing Treg cell production (128).

### Effects of MDS-MSCs on neutrophils

Besides inhibiting neutrophil respiratory burst, BM-MSCs can also prolong neutrophil survival and thus play an anti-inflammatory role, which involves the IL-6 and STAT-3 signaling pathways. Neutrophils in patients with MDS appear to be significantly defective, resulting in an inability to perform anti-infective functions. For example, Cao et al. demonstrated defective chemokine-dependent chemotaxis and enhanced degranulation associated with the expression of key structural proteins, such as DOCK8, Cdc42, and Rac1, in neutrophils derived from 12 patients with MDS (135). This finding confirms that in these patients, neutrophil defects prevent it from migrating effectively, and this may lead to altered microenvironment and ultimately adverse clinical outcomes. *In vitro*, MSC-MVs preferentially promote granulocyte expansion (56).

## Conclusions

BM-MSCs are involved in immunoregulation; MDS has various pathogenetic mechanisms, among which BM-MSCs play a significant role in the immune-related pathogenesis. In addition to their immunomodulatory properties, BM-MSCs have low immunogenicity (136), rendering them useful for the treatment of immunological disorders (137). The advantages of exosomes in disease treatment include their low toxicity, biological barrier penetrability, stability, and biocompatibility (138). In addition, the advantages of using exosomes for the treatment of hematological malignancies are reflected in that, firstly, it is a non-invasive treatment method, and secondly, its mechanism can protect the exosome contents from the degradation of nucleases and proteases. Exosomes can be used as liquid biopsies to diagnose and stage disease and for monitoring disease progression and response to treatment (139). In addition, drug-loaded exosomes can bind to adjacent cell membranes to transport drugs to target cells. Another advantage of drug-loaded exosomes is that they prevent rapid clearance by the phagocytic system and increase the duration of action of the drug *in vivo*. As an endogenous component of the body, exosomes are expected to become biological carriers for drug transport (140–142). In addition to monoclonal antibody-based therapy against immune cells, an BM-MSC-based therapeutic strategy against immunoregulatory targets will serve as a novel option for the treatment of patients with MDS.

## Author contributions

All authors listed have made a substantial, direct and intellectual contribution to the work, and approved it for publication.
